# Defenders of the Transcriptome: Guard Protein-Mediated mRNA Quality Control in *Saccharomyces cerevisiae*

**DOI:** 10.3390/ijms251910241

**Published:** 2024-09-24

**Authors:** Luisa Querl, Heike Krebber

**Affiliations:** Abteilung für Molekulare Genetik, Institut für Mikrobiologie und Genetik, Göttinger Zentrum für Molekulare Biowissenschaften (GZMB), Georg-August Universität Göttingen, 37077 Göttingen, Germany; luisa.querl@uni-goettingen.de

**Keywords:** RNA-binding proteins, mRNA export, mRNA surveillance, quality control, SR proteins, post-transcriptional modifications

## Abstract

Cell survival depends on precise gene expression, which is controlled sequentially. The guard proteins surveil mRNAs from their synthesis in the nucleus to their translation in the cytoplasm. Although the proteins within this group share many similarities, they play distinct roles in controlling nuclear mRNA maturation and cytoplasmic translation by supporting the degradation of faulty transcripts. Notably, this group is continuously expanding, currently including the RNA-binding proteins Npl3, Gbp2, Hrb1, Hrp1, and Nab2 in *Saccharomyces cerevisiae*. Some of the human serine–arginine (SR) splicing factors (SRSFs) show remarkable similarities to the yeast guard proteins and may be considered as functional homologues. Here, we provide a comprehensive summary of their crucial mRNA surveillance functions and their implications for cellular health.

## 1. Introduction

To ensure the export of only correctly generated mRNAs, a series of quality-control mechanisms occurs in the nucleus. As different mRNA features are essential for trouble-free gene expression, pre-mRNAs are already surveilled at different time points during transcription. At an early stage of transcription, when approximately 13–20 nucleotides are transcribed, various proteins mediate 5′-capping. This process ensures that a cap-binding complex (CBC)-bound 7-methylguanosine (m^7^G) cap is synthesized, which helps pre-mRNAs withstand nucleolytic attacks [1,2]. Subsequently, noncoding introns are excised from the open reading frame (ORF) by the spliceosome [3]. Importantly, for splicing to work efficiently, a properly capped 5′-end of the pre-mRNA must exist [4], already revealing the importance of functional quality-control mechanisms. Upon arrival of the RNA polymerase II (RNAPII) containing transcription machinery at the 3′-end of the pre-mRNA, the termination machinery is recruited, and the transcript is released from the DNA. Correct termination requires the cleavage and polyadenylation complex (CPF-CF), which is directed by cis-acting elements located in the 3′ untranslated region (UTR) [5]. If termination fails, transcripts are terminated downstream of the CPF-CF site, mostly by the Nrd1, Nab3, and Sen1 (NNS)-mediated termination system, which leads to the elimination of the readthrough transcript [6,7,8]. Finally, a poly(A) tail is attached to the 3′-end of the mRNA to protect the transcript from exonucleolytic attacks from this RNA end [9].

**Figure 1:**
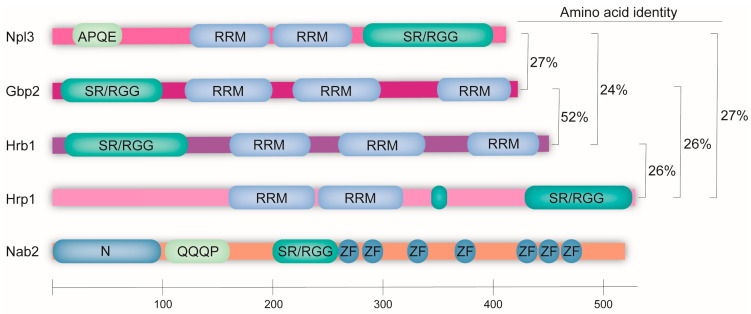
The SR/RGG-rich guard proteins share domains and participate in several quality-control steps during pre-mRNA maturation. The domain structure of the yeast guard proteins and their level of amino acid identity is shown. The five homologous proteins all contain a serine–arginine or arginine–glycine–glycine-rich domain (SR/RGG) that is important for shuttling and protein modifications. They also contain RNA-binding domains, either as RNA-recognition motifs (RRMs) or as a zinc-finger domain (ZF). Npl3 additionally contains a domain with the repeatedly occurring amino acid residues APQE and Nab2, and it contains a Q-rich domain at the N-terminus. Currently, both domains are of unknown function.

These regulatory steps are overseen by a group of proteins called the guard proteins (Table 1). In the context of *Saccharomyces cerevisiae*, several guard proteins have been discovered, including Npl3, Gbp2, Hrb1, Nab2, and the recently identified Hrp1. Each of these proteins is characterized by the presence of several RNA-binding motifs, notably the RNA-recognition motif (RRM) or zinc-finger (ZF) domain, as well as a serine–arginine/arginine–glycine–glycine (SR/RGG) domain. Among the guard proteins, Npl3 contains the most canonical SR/RGG domain at its C-terminus, along with two central RRMs and an N-terminal APQE domain still awaiting characterization (Figure 1) [10]. Interestingly, the two RRMs are not equivalent, as the second RRM is able to form stronger interactions with guanine/uracil (G/U)-rich sequences on RNA [11]. The paralogues Gbp2 and Hrb1 both feature an N-terminal SR/RGG domain mainly containing SR repeats and three RRMs each. While RRM1 and RRM2 preferentially recognize RNAs containing the core motif GGUG, the atypical RRM3 at the C-terminus does not interact with nucleic acids but is crucial for the interaction with the THO/TREX complex [12]. Hrp1 exhibits a C-terminal SR/RGG domain rich in single-serine and arginine residues, in addition to two centrally located RRMs. As evident by the lack of common amino acid identity with the other guard proteins, Nab2 varies in its domain composition (Figure 1). Instead of RRMs, it contains seven ZF motifs within its C-terminal ZF domain [13]. The ZF domain is critical for its interaction with poly(A) RNA, with ZF 5-7 being crucial for this function [13]. Additionally, Nab2 harbors an N-terminal domain essential for the nuclear export of poly(A) RNAs and Nab2 itself, a nonessential glutamine (Q)-rich domain of currently unknown function, and the SR/RGG domain, which is important for the reimport of Nab2 [13]. These structural features play a crucial role in facilitating the interactions of all guard proteins with the nascent mRNA during the various stages of its maturation [14].

**Figure 2:**
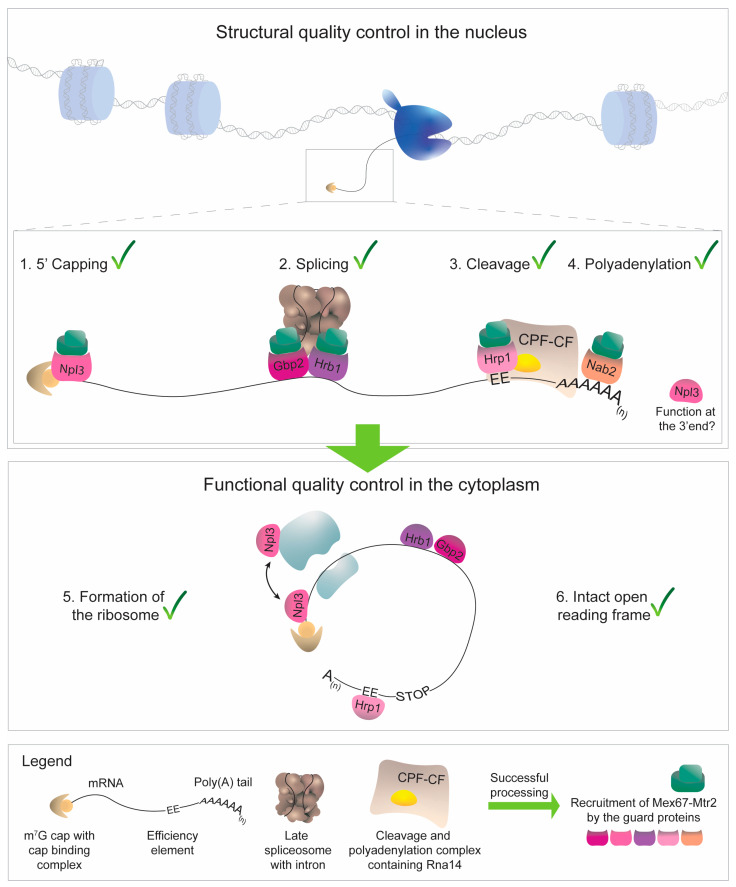
The stepwise mRNA maturation is controlled by the guard proteins. 5′ capping is recognized by Npl3, which identifies the cap-binding complex (CBC)-bound m7G cap. Completed splicing is recognized by the interaction of Gbp2 and Hrb1 with the late spliceosome. Then, 3′ cleavage is monitored by Hrp1, which detects the binding of the CPF-CF protein Rna14. Nab2 recognizes and binds the poly(A) tail at the 3′ end. An involvement of Npl3 in 3′ processing has been proposed. However, the underlying mechanism is currently unclear. In the cytoplasm, the guard proteins support the formation of the ribosome and their positioning in the proper context signals the presence of an intact open reading frame.

At their core, all of the guard proteins share a common function as retention factors for mRNAs, which they conduct until the recruitment of the export receptor Mex67-Mtr2 (short Mex67) after the successful completion of a step in the mRNA-maturation pathway (Figure 2) [15,16,17,18]. Generally, the more Mex67 is bound to an mRNA, the more it is shielded from the hydrophobic interior of the nuclear pore complex (NPC), enabling better passage into the cytoplasm [19]. Coverage with the export receptor is controlled by the interaction of the guard proteins with Mlp1, the gatekeeper of the NPC. In this way, Mlp1 prevents the passage of immature pre-mRNAs when not sufficiently Mex67-covered. All of the guard proteins are nuclear at a steady state, and their export depends on active transcription and the export of mRNAs in general. Upon correct maturation, they accompany the mRNA into the cytoplasm, where translation occurs. Here, many of them serve a second purpose in cytoplasmic mRNA surveillance, as most are either involved in translation or participate in nonsense-mediated decay (NMD) [20,21,22]. The release of the guard proteins from mRNA occurs through distinct mechanisms. For Npl3, Gbp2, and Hrb1, dissociation is primarily facilitated through binding with the import receptor Mtr10, while for Hrp1 and Nab2, dissociation occurs via interaction with the import receptor Kap104 [23,24,25].

**Figure 3:**
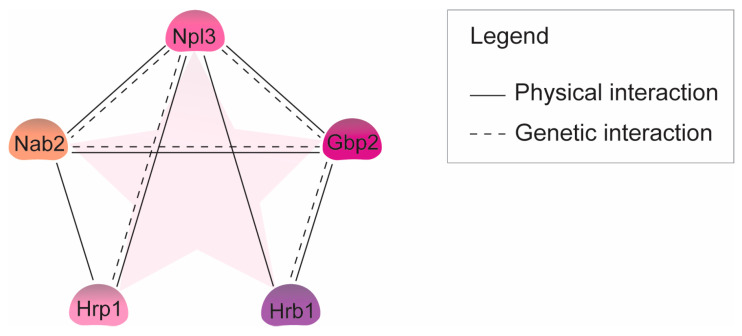
The guard proteins interact with each other. Interaction map showing physical and genetic interactions between each of the guard proteins. Known physical interactions are indicated by a solid line, while genetic interactions are visualized with a dashed line.

All currently known guard proteins share the common trait of interacting physically and genetically with the export receptor Mex67 and the NPC gatekeeper Mlp1 [16,18,26,27,28,29]. Additionally, they exhibit mutual physical and genetic interactions among themselves (https://www.yeastgenome.org/ (accessed on 1 August 2024)) (Figure 3). While typically responsible for retaining only faulty transcripts, it is notable that the overexpression of any guard protein results in the widespread retention of poly(A)+ RNAs in the nucleus. This phenomenon is likely due to excessive binding of the proteins to the same transcripts, possibly overwhelming the capacity of Mex67 for coverage or not being situated in the correct context to allow Mex67 binding, therefore leading to retention, which proves detrimental to cellular health [16,27]. Lastly, a shared characteristic among the guard proteins is their interaction with the cellular degradation machinery, ensuring the timely elimination of faulty transcripts [15,16,17,18,30].

## 2. Maturation of Pre-mRNAs

mRNAs are continuously synthesized through transcription by RNAP II from their 5′- to 3′-ends, with the initial modification being mRNA capping. During transcription initiation, an inverted 7-methylguanosine (m7G) cap is synthesized to protect the 5′-end from exonucleolytic degradation, promote proper splicing and nuclear export, and facilitate efficient translation initiation [1,2,31]. The capping process involves three key enzymes: The 5′ RNA triphosphatase Cet1 converts the 5′ triphosphate into a diphosphate, the RNA guanylyltransferase Ceg1 adds a guanosine-7 cap, and the methyltransferase Abd1 methylates the cap [32,33,34,35]. The 5′ cap is immediately recognized and bound by the CBC, which facilitates subsequent processing steps, including splicing, nuclear export, and translation initiation in the cytoplasm [36,37].

Similar to those in higher eukaryotes, pre-mRNAs in yeast may contain noncoding introns, which are excised during splicing to create a mature ORF. The splicing process is mediated by the spliceosome, a large ribonucleoprotein complex composed of small nuclear RNA (snRNA) and protein counterparts. Splicing factors are recruited throughout transcription elongation to determine the proper splice sites and facilitate the assembly of the spliceosome [3,38,39]. The yeast spliceosome comprises five snRNAs, termed U1, U2, U4, U5, and U6, along with approximately 70 binding proteins, which together form small nuclear ribonucleoprotein particles (snRNPs) [40,41].

Once splicing is completed, the mature mRNA undergoes further processing steps, including 3′ end cleavage and polyadenylation, which may occur in close proximity to the nuclear pores [42]. This process is crucial for stabilizing the mRNA, regulating its nuclear export and facilitating efficient translation in the cytoplasm. For 3′ polyadenylation and cleavage, numerous proteins form the cleavage and polyadenylation complex (CPF-CF). Specific sequences within the 3′ UTR of the pre-mRNA serve as recognition sites for the cleavage and polyadenylation machinery. Sequences such as the positioning element (PE) or efficiency element (EE) upstream of the cleavage site help tether the complex to the mRNA, while downstream sequence elements flanking the cleavage site may help make the poly(A) site (PAS) more accessible. The recognition of the PAS initiates the cleavage of the pre-mRNA by the endonuclease Ysh1, leaving a free 3′ hydroxyl group. Finally, the poly(A) polymerase Pap1 adds a 60–80 nucleotide long poly(A) tail to the 3′-end of the mRNA to protect it from exonucleolytic attacks from this RNA-end [8,9,43]. The mature mRNA is then ready for export from the nucleus to the cytoplasm, where it can be translated into a functional protein.

### 2.1. Guard Protein-Mediated 5′ Capping Requires Npl3

**Figure 4:**
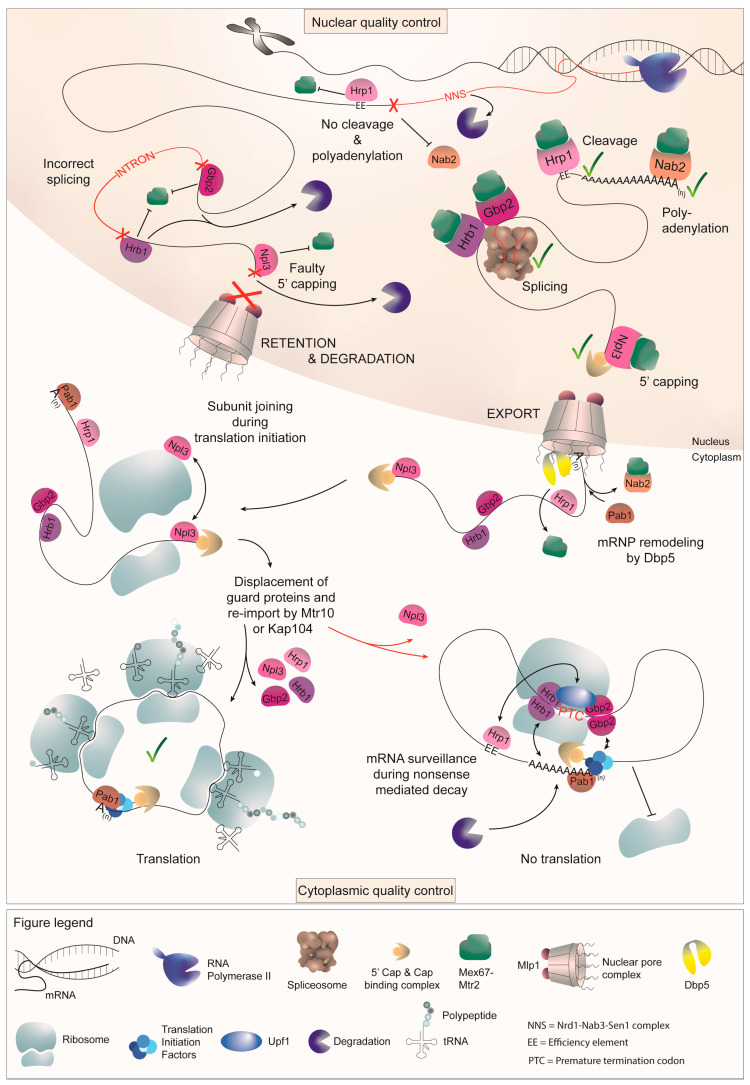
The SR-rich guard proteins mediate mRNA quality control in the nucleus and the cytoplasm. In the nucleus, the SR/RGG motif containing guard proteins is loaded onto the nascent pre-mRNA co-transcriptionally. Npl3 is recruited during early transcription and surveils 5′ capping, Gbp2 and Hrb1 are loaded during splicing and control proper intron excision, while Hrp1 and Nab2 bind during 3′ processing and surveil cleavage and polyadenylation, respectively. All of the guard proteins recruit the export receptor Mex67-Mtr2 upon correct mRNA processing and their coverage signals the export competence of the mRNP to the NPC gatekeeper Mlp1. While Mex67-Mtr2 is depleted from the mRNA by the RNA helicase Dbp5 in the cytoplasm, all of the guard proteins except for Nab2 remain bound to the transcript to facilitate further tasks during translation. Nab2 is replaced by the poly(A) tail-binding protein Pab1. Npl3 assists in monosome formation during translation initiation, where it may assist in subunit joining through interactions between mRNA-bound Npl3 and Npl3 bound to the large ribosomal subunit. Gbp2 and Hrb1 assist in nonsense-mediated decay by signaling the presence of a PTC to the ends of the folded mRNA, thereby inhibiting further translation initiation and recruiting cytoplasmic decapping and degradation factors. Hrp1 may signal the presence of a PTC by associating with the 3′ end of an mRNA.

The process of 5′ capping is monitored through the shuttling guard protein Npl3, which is recruited to the nascent pre-mRNA by RNAP II during early transcription [44]. It serves as an adaptor for Mex67 and modulates the switch between export and degradation, surveilling the proper capping of transcripts due to its proximity to the 5′ end. Notably, Npl3 associates with pre-mRNAs bound by the 5′ end degradation factor Rai1 and recruits the export receptor Mex67 only after successful capping. In that case, the CBC binds the 5′ end of the mRNA, and Rai1 dissociates, leaving Npl3 to be able to recruit the export receptor. However, in the absence of correct CBC attachment, Npl3 fails to sense proper capping, allowing for the 5′ exonuclease Rat1 to interact with Rai1 and degrade the faulty transcript. In cases where immediate degradation is not possible, Npl3 retains the transcript in the nucleus, while the absence of Npl3 can lead to the leakage of uncapped transcripts into the cytoplasm (Figure 4) [15].

Unlike other Mex67-recruiting proteins that remain in the nucleus and are displaced from the mRNA prior to export, the guard proteins continue to stay bound to the mRNA after export. They shuttle with the messenger ribonucleoprotein (mRNP) into the cytoplasm, enabling them to fulfill additional tasks. Likewise, Npl3 also participates in the nuclear export of large ribosomal subunits, in addition to its function in mRNA surveillance and export [45]. Similar to its attachment to mRNAs, which persists even after export, Npl3 remains bound to the large ribosomal subunit after shuttling as well. Being bound to both, the mRNA on which the small subunit is positioned at the AUG start codon and the large ribosomal subunit, Npl3 further assists the ribosomal monosome formation during translation initiation. As the small 40S ribosomal subunit first binds to the mRNA upon translation initiation, the 60S subunit-bound Npl3 contributes to successful subunit joining by self-dimerizing with its counterparts bound to the mRNA (Figure 4) [21]. This cytoplasmic function of Npl3 promotes the assembly of the translation machinery only onto properly capped mRNAs. This is crucial for further RNA protection but also for subsequent translation, because circularized RNAs are more efficiently translated, as the ribosomal subunits are recycled to the 5′-end for another round of translation after reaching the stop codon on the mRNA 3′ end [46,47]. In this way, Npl3 helps to make translation as efficient as possible, since mRNAs that have failed quality control are rather degraded than those allowed to occupy the translation machinery.

### 2.2. Intron Splicing Is Surveilled through the Guard Proteins Gbp2 and Hrb1

Among the guard proteins, Gbp2 and Hrb1 stand out as key players in regulating proper splicing. During splicing, these paralogues are co-transcriptionally recruited to pre-mRNAs by the THO complex [24,48,49,50]. They function as a switch between export and degradation, as they retain transcripts in the nucleus until splicing is completed successfully or until a faulty transcript is marked for degradation. Hence, Gbp2 and Hrb1 contact the spliceosome in late splicing and either recruit the export receptor Mex67-Mtr2 to signal export competence or recruit the TRAMP complex, which subsequently assists in the degradation of the transcript [18]. The TRAMP complex, comprising the RNA helicase Mtr4, zinc-finger mRNA-binding subunits Air1 or Air2, and either Trf4 or Trf5 polymerases, assists in degrading transcripts by adding a short oligo(A) tail to the 3′ end, marking them for nuclear exosome-mediated degradation [51,52]. Conversely, coverage of the guard proteins with Mex67 signals proper splicing to the NPC gatekeeper protein Mlp1, allowing their export into the cytoplasm as a result of correct processing. When Gbp2 and Hrb1 are absent from cells, unspliced transcripts can be observed leaking into the cytoplasm [16,18].

Gbp2 and Hrb1 remain bound to spliced mRNAs until translation, where they assist in the degradation of faulty transcripts containing a PTC [20]. Both proteins exhibit a preference for binding to the 5′ end of mRNAs, presumably due to the prevalence of yeast introns in this region [53,54]. Upon recognition of a PTC, a complex containing Upf1-2-3 is formed and Gbp2 and Hrb1 facilitate the remodeling of the RNP. The mRNA is circularized to ensure fast and efficient rounds of translation, and upon PTC detection, Gbp2 and Hrb1 are able to restructure the mRNA, conveying the PTC signal to both ends of the transcript, ultimately triggering RNA degradation. Their ability to form dimers with themselves, as well as with each other, suggests that their interactions may bring the PTC recognition site close to the transcript ends through necessary mRNA folding. Additionally, interactions with 5′ or 3′ end-associating proteins such as Pab1 or some of the transcription initiation factors (TIFs) may further assist the RNA remodeling. This way, further translation initiation is inhibited and degradation is initiated (Figure 4) [20].

While both Gbp2 and Hrb1 mediate translational inhibition, the degradation process differs between them. Hrb1 recruits the decapping factor Dcp1 [20], which allows the degradation of the aberrant mRNA by the cytoplasmic 5′-3′ exonuclease Xrn1 after decapping [55,56]. The minor degradation pathway from the 3′ end, on the other hand, is propagated by Gbp2, which recruits the Ski complex and thus designates the mRNA for degradation by the cytoplasmic exosome [20].

### 2.3. Proper 3′ Cleavage Is Monitored by Hrp1

Hrp1 has recently emerged as the candidate to fill the role of a guard protein for 3′ cleavage, completing the set of guard proteins for each primary mRNA-processing step. In the nucleus, Hrp1 binds to the UAUAUA-containing efficiency element (EE) located in the 3′ UTR of the mRNA and shuttles into the cytoplasm along with the transcript [57,58,59]. It is a component of the cleavage and polyadenylation factor (CPF) and cleavage factor (CF) complex, which is crucial for the 3′ end cleavage and release of pre-mRNA from RNAPII. Hrp1 binds to the EE located upstream of the mRNA cleavage site, recruits the export receptor Mex67-Mtr2 upon correct cleavage, and also interacts with the nuclear gatekeeper Mlp1 [9,27,60]. Interestingly, it is the only component of the CPF-CF that shuttles into the cytoplasm alongside transcripts [57,58,61]. Hrp1 is able to sense successful cleavage through its contact with Rna14, which is a scaffold component of the CPF-CF and the primary interaction partner for Hrp1. This contact allows Mex67 to bind the guard protein, while the absence of the Rna14-Hrp1 interaction, possibly due to an improper assembly of the CPF-CF and, therefore, readthrough of the termination site, leads to the retention of the transcript [27]. Interestingly, Hrp1 stands out as the only guard protein lacking direct contact with the nuclear-degradation machinery. Instead, contact with nuclear-degradation factors depends on the NNS fail-safe termination system, which detects transcription readthrough and initiates the subsequent degradation of the faulty transcript [6]. The NNS complex consists of two RNA-binding proteins, Nrd1 and Nab3, which specifically recognize the RNA sequences GUAA/G and UCUU downstream of the CPF-CF termination site [62,63]. The RNA-DNA helicase Sen1 serves as the third component of the complex, likely recruited by Nab3 [64]. Together, the NNS complex facilitates termination by displacing RNAPII, and the interaction of Nrd1 with the exosome and the TRAMP component Trf4 results in the recruitment of the degradation machinery [65,66].

In the absence of Hrp1, elongated transcripts are no longer retained in the nucleus and leak into the cytoplasm [27]. Most importantly, mutation of *HRP1* results in 3′ extended transcripts for the majority of all mRNAs already after a 1 h shift to the non-permissive temperature, suggesting that Hrp1 has a broad function as a nuclear and cytoplasmic quality-control factor. Again, such elongated transcripts are suboptimal for translation, as long 3′ UTRs lower the half life of an mRNA and may trigger NMD, as the ribosome recycling process becomes more difficult. In addition to its role in nuclear quality control, Hrp1 has been implicated in the process of NMD. Hrp1 has been reported to interact with the NMD factor Upf1, and it supposedly associates with so-called downstream sequence elements (DSEs) [22]. Initially, these sequences were believed to be located in coding regions, where they were bound by RNA-binding proteins that were subsequently replaced by the translating ribosome. In the presence of a PTC, the presence of these proteins would then trigger NMD. Although this model has not been extensively explored due to the fact that the DSE sequences have proven to be highly degenerate and because alternative NMD models in yeast have emerged, the notion that PTCs might be recognized through marks downstream of the termination codon remains relevant in current hypotheses. Transcriptome-wide studies have revealed that Hrp1 is widely present in most mRNAs [27]. Thus, the EE might be the main region to which Hrp1 binds in the nucleus and remains bound until translation.

### 2.4. Nab2 Surveils the Proper Attachment of a Poly(A) Tail

In yeast, Nab2 is the nuclear poly(A) tail-binding protein that monitors the tail length and interacts with the export receptor Mex67 to facilitate mRNA transport through the NPC [30,67,68]. Similar to the other guard proteins, Nab2 also interacts with Mlp1 at the NPC, creating another checkpoint for nuclear export [68]. Moreover, mutations in Nab2 have been observed to result in the leakage of faulty transcripts into the cytoplasm [16]. Notably, Nab2 is distinct among the guard proteins as it is displaced from the mRNA at the cytoplasmic side of the NPC by the DEAD-box RNA helicase Dbp5 [69]. Consequently, Nab2 is the first guard to leave the mRNA, which is also reflected in its absence from polysomes [50]. While the other guards remain bound until translation commences and, in the case of Gbp2, Hrb1, and Hrp1, are also involved in NMD, Nab2 departs prior to the start of translation. Nab2 is most likely replaced by Pab1, which enables the circularization of the mRNA, occurring through the interaction of Pab1 and the 5′ cap-binding complex, including eIF4G [70]. The circularization of the transcript is thought to enhance ribosome cycling for more efficient translation [71].

Furthermore, beyond its role in maintaining optimal poly(A) tail length, Nab2 likely serves an additional function in facilitating the formation of correctly folded, export-competent mRNPs. Due to its documented propensity for dimerization via the ZF domain and reported binding along the entire transcript length, it is hypothesized that Nab2 contributes to the correct folding of mRNPs [72,73]. Moreover, mutations within the ZF domain have been shown to promote an accelerated disassembly of the mRNP by Dbp5 in the cytoplasm, presumably due to substandard packaging [74].

### 2.5. Npl3 Participates in 3′ End Quality Control

Interestingly, Npl3 may emerge as an additional key player in nuclear 3′ end quality control, fulfilling a function during the final stages of transcription. It appears to rival several 3′ end processing factors for pre-mRNA binding, offering protection against premature termination and facilitating the coordination of transcription termination and the packaging of finalized transcripts for export [11]. Notably, the functional dynamics of Npl3 are influenced by phosphorylation mediated by CKII during these crucial stages, which may finetune the termination process by enabling the binding of termination factors through a reduced RNA-binding capacity of Npl3 [75,76]. Additionally, Rat1 and Rai1, which cooperate with Npl3 during 5′ quality control in order to ensure proper degradation, have been implicated in poly(A)-dependent transcription termination as well [32,77]. Moreover, the absence of Npl3 leads to a widespread 3′ extension of transcripts transcribed by RNAPII, indicating the possibility of anomalies in pre-mRNA packaging events that may lead to termination readthrough. This widespread readthrough has also been linked to the downregulation of neighboring genes, underscoring the significance of Npl3 in maintaining proper termination and gene expression [78]. While the precise mechanism of the involvement of Npl3 in nuclear 3′ end quality control remains to be fully elucidated, these observations strongly indicate a role for Npl3 in this process.

## 3. The Guard Proteins Are Subjected to Post-Translational Modification

Post-translational modifications (PTMs) play a crucial role in modulating the functions and activities of a plethora of proteins. Modifications such as methylation or phosphorylation are also observed across the spectrum of the previously described guard proteins. Although numerous sites for a variety of PTMs have been reported for all of them, our understanding of the functional implications of many of these modifications remains incomplete. Currently, the best-described modifications are methylation and phosphorylation.

Among the guard proteins, Npl3 stands out as the cornerstone of our understanding of PTMs. As such, unphosphorylated Npl3 is recruited co-transcriptionally by the phosphorylated Ser2 of RNAPII, promoting RNAPII elongation and preventing premature termination by competing with Rna15 for RNA binding at the 3′ end. Release of Npl3 from the CTD is achieved through phosphorylation by casein kinase 2 (CK2) at several serine residues, most of which are located within the SR/RGG domain [75]. In the nucleus, Glc7-mediated dephosphorylation promotes the association of Npl3 with the export receptor Mex67 [79]. It has been suggested that Sky1-mediated phosphorylation aids Npl3 dissociation from mRNA in the cytoplasm, with the process resembling phosphorylation in the nucleus, as many of the same residues targeted by CK2 in the nucleus are also targeted by Sky1 [75,80]. However, gradient analysis of polysomes revealed that the import receptor Mtr10 is involved in the dissociation of Npl3 from the mRNA [50]. This interaction with the import receptor then enables phosphorylated Npl3 to reimport into the nucleus [81]. Additionally, Npl3 undergoes methylation by the methyltransferase Hmt1, a modification potentially regulating its export by modulating interactions with nuclear proteins and promoting splicing [80]. Regulation of methylation may be facilitated by Air1 and Air2 through Hmt1 inhibition [82]. While numerous phosphorylation and methylation sites have been documented for Npl3, reports also suggest succinylation and acetylation, although their functional significance remains elusive [83].

For the remaining guard proteins, methylation predominates in our understanding of PTMs, as all of them are also methylated by Hmt1. Consequently, Hmt1-catalyzed methylation plays a significant role in modulating the function of Hrp1 [80]. Interestingly, methylated Hrp1 binds to the EE of mRNA with the same efficiency as its unmethylated counterpart, but the presence of RNA itself inhibits Hrp1 methylation, particularly if the RNA displays a strong binding affinity to Hrp1 [84]. Deletion of Hmt1 itself results in only a minor growth defect [85] but leads to substantial alterations in Hrp1 binding to the gene. Thus, methylation appears to be crucial for ensuring the proper association of Hrp1 with genomic targets [86]. Furthermore, methylation reportedly facilitates the export of Hrp1 from the nucleus [87]. This export mechanism may be intertwined with the methylation state of Npl3, as the exchange of methylated arginine residues in Hrp1 is insufficient to fully block export, but instead, methylated Npl3, as part of the mature mRNP complex, likely plays a pivotal role [83]. In addition to multiple reported methylation sites, a large number of Hrp1 phosphorylation sites have been identified [83,88,89,90]. Moreover, Hrp1 is subject to sumoylation, succinylation, acetylation, and ubiquitylation, expanding the repertoire of PTMs possibly implicated in its regulation [89,91,92,93].

The poly(A)-binding protein Nab2 undergoes arginine modification by Hmt1 as well, with methylation primarily being focused on the SR/RGG domain. Similar to Npl3 and Hrp1, Nab2 methylation is required for the export of the protein, indicating a conserved role of methylation in regulating the localization of guard proteins [30]. Moreover, the absence of Hmt1 leads to significant alterations in the genome-wide binding profile of Nab2, underscoring the importance of methylation in modulating the Nab2 function [86]. Alongside reported methylation sites, several phosphorylation sites have also been identified for Nab2 [83,89,90].

In contrast to Npl3, Hrp1, and Nab2, the export of the splicing guards Gbp2 and Hrb1 appears to be unaffected by a lack of methylation, although methylation sites have been reported for both proteins [49,83,87]. For Gbp2, methylation by Hmt1 at seven methylation sites has been documented, yet this modification does not significantly impact protein–protein interactions, as evident by an unchanged binding between Gbp2 and Npl3 [94]. Additionally, several phosphorylation sites have been described for both Gbp2 and Hrb1 [89,90].

The complex interplay of PTMs among the guard proteins underscores the intricacy of the mRNA export machinery, with methylation and phosphorylation emerging as crucial regulatory mechanisms essential for proper protein shuttling. However, the diverse responses of the guard proteins to these modifications highlight the nuanced dynamics of mRNA metabolism, with many aspects still remaining unclear. While research has primarily focused on methylation and, in the context of Npl3, phosphorylation, exploring different modifications could also present an interesting topic for investigation. Unraveling the complex network of PTMs may lead to a more comprehensive understanding of the mRNA life cycle and its regulation.

## 4. Human Guard Proteins

Potential homologues of the SR/RGG containing guard proteins also exist in humans. Notable human counterparts can be found within the large group of heterogenous nuclear ribonucleoproteins (hnRNPs), some of which shuttle with the mRNA into the cytoplasm [95]. Among the class of RNA-binding proteins, those containing an RGG domain are the second-most common [96,97]. In humans, the yeast guards are probably most closely related to the shuttling SR-motif containing RNA-binding proteins SRSF1-12, several of which shuttle with the mRNA from the nucleus into the cytoplasm and interact with the Mex67-Mtr2 homologue TAP-p15 [98,99,100,101]. The human SR proteins are structurally related, as all of them contain at least one RRM in addition to the C-terminal SR domain [102]. Currently, proteins are categorized as canonical SR proteins if they meet specific criteria: They have to possess one or two conserved RRMs and a C-terminal SR domain containing at least 50 amino acids with an SR content of more than 40% [103]. All of the human SR proteins are predominantly nuclear, but SRSF1, 3, 4, 6, 7, and 10 are known to shuttle into the cytoplasm, with SRFS1, 3, 7, and 10 shuttling at a faster rate than SRSF4 and 6 [104,105,106,107]. SRSF2 and 5 have also been reported to shuttle in cells that are not yet differentiated [108]. SR proteins are mainly involved in the highly intricate process of alternative splicing, as they often recruit other splicing factors to pre-mRNAs. They also appear to be essential for other processes such as transcriptional activation, NMD, mRNA export, or translation, but the exact involvement in these processes remains to be elucidated [109,110,111]. Arginine methylation has been identified as a regulator of the nucleo-cytoplasmic distribution of certain SR proteins [100,112]. Moreover, SRSF3 has been implicated in the degradation of intronless viral transcripts by recruiting the nuclear exosome through interactions with both the exosome and its adapter complex, NEXT [113]. These discoveries highlight striking parallels to the functions of the yeast guard proteins. However, much remains unknown regarding the specific functions of SR proteins, particularly in the context of mRNA quality control. Implementing systematic leakage studies of faulty transcripts could represent a promising avenue for further investigation into the intricacies of their quality-control functions. Dysregulation of SR/RGG proteins has been linked to various human diseases, including cancer and neurodegenerative diseases [114,115,116,117,118,119,120,121] (https://www.cbioportal.org/ (accessed on 1 August 2024)). In yeast, the lack of classical SR proteins meeting the human classification criteria can be attributed to the inexistence of alternative splicing. Thus, while there are no direct homologies in humans, considering the functional similarities, the notion of human SR proteins serving as potential mRNA quality-control factors and guard proteins for transcript maturation appears plausible.

While Npl3 shows a direct relation to the human SR proteins SRSF4, SRSF5, and SRSF6, the other yeast guards have more close orthologues among the hnRNPs. Nab2, for instance, is related to the human hnRNP ZC3H14, which plays a role in controlling poly(A) tail length in neuronal cells and contains a conserved zinc finger motif [122]. ZC3H14 has even been shown to be able to functionally substitute for Nab2 in Drosophila neurons, highlighting their functional similarity in regulating RNA processing [123]. The closest orthologue for Hrb1 is human MYEF2, a nuclear protein with three RNA-recognition motifs (RRMs) involved in neuronal differentiation [124]. Similarly, Gbp2 is most closely related to human HNRNPM, a paralog of MYEF2, which binds pre-mRNAs and participates in splicing [125]. Additionally, Hrp1 has orthologs in human hnRNPs such as HNRNPDL, HNRNPA1, HNRNPD, HNRNPA3, and HNRNPAB, further emphasizing the functional parallels between these proteins.

Another intriguing perspective arises from the possibility that the yeast guard proteins could serve as precursors to the human exon junction complex (EJC). In higher eukaryotes, the multi-protein EJC plays a pivotal role in mRNA metabolism. During splicing, EJCs associate with mRNA approximately 20–24 nucleotides upstream of the exon-exon junction, independent of sequence, and accompany the mRNA throughout its lifecycle [126]. Interacting with a myriad of proteins, EJCs influence almost every step of gene expression [127]. Notably, the EJC shuttles with the mRNA into the cytoplasm and is only displaced by the translating ribosome [128]. Moreover, its presence downstream of a premature stop codon typically triggers NMD [129,130]. Since the yeast guard proteins also interact with mRNA in a similar manner, it is intriguing to speculate that they might be precursors of EJCs in higher eukaryotes. One of the EJC key components, RBM8A (also known as Y14), further exhibits remarkable structural similarities to the smallest SR protein SRSF3. RBM8A not only contains a central RRM but also a C-terminal RS-rich, SR protein-like sequence. This sequence, which undergoes modifications such as phosphorylation and methylation, is crucial for the protein’s localization within the cell [131,132]. EJCs have been reported to interact with a multitude of SR proteins, and their interaction even stabilizes the association of SR proteins with poly(A)+ RNA [133]. The interaction of EJCs and SR proteins likely enhances correct mRNP packaging, showing an additional parallel to how the yeast guard proteins contribute to proper mRNP processing. Further exploration of this concept could shed light on the functional conservation of mRNA quality-control systems.

Undoubtedly, the transcriptomic organization in humans is considerably more complex than in yeast. In yeast, despite high transcriptional activity, intron-containing genes are relatively rare (<5%), and introns themselves are small, with an average length of 154 base pairs for non-ribosomal protein-coding genes [134,135]. Moreover, most intron-containing genes have only one intron, with very few exceptions having more [135]. Alternative splicing is also uncommon, and while yeast does have many long non-coding RNAs, their numbers are modest compared to humans. In contrast, human RNA processing involves a vast majority of intron-containing genes (>97%), with introns often spanning several thousand base pairs and a median length of over 1700 bp for protein-coding genes [136]. More than 40% of human genes contain at least three introns [137], and alternative splicing is a widespread phenomenon, affecting approximately 95% of multi-exon genes and generating numerous mRNA and protein isoforms [138]. Additionally, only 1–2% of the human genome consists of coding DNA, while there are over 60,000 lncRNAs alone [139] and there are multiple RNA species that are lacking in yeast, such as miRNAs and siRNAs. This complexity demands a more intricate RNA quality control system, which must also regulate the abundant ncRNAs. Despite these differences, the simpler RNA surveillance mechanisms in yeast may provide valuable insights. Yeast guard proteins could very well represent evolutionary precursors to analogous human RNA-binding proteins, such as SR proteins, hnRNPs, and components of the EJC. Understanding the fundamental processes in yeast may guide future research into uncovering analogous pathways in humans, offering directions for elucidating how RNA integrity is maintained in more complex transcriptomes.

## 5. Outlook

RNA transcripts come in various forms, and mRNAs represent just one category within the vast spectrum of the transcriptome. In addition to coding RNAs, many noncoding (nc)RNAs exist. As such, recent studies have shown that antisense (as)RNAs boost the expression of their sense mRNA counterpart by forming a double strand [140]. The involvement of guard proteins in controlling the process of the double-strand formation remains to be elucidated. Additionally, other non-coding RNAs, such as the previously mentioned snRNAs, which are important for splicing, but also snoRNAs, tRNAs, or ribozyme RNAs, such as the telomerase, may be influenced by guard protein-mediated quality control. All of these transcripts depend on properly processed 3′ and 5′ ends as well, but so far, limited information is available about the surveillance of these mechanisms. Interestingly, the maturation pathway of ncRNAs such as snRNAs or the telomerase involves nucleo-cytoplasmic shuttling [37,141,142], and both of these transcripts also interact with some of the guard proteins [53,54]. Therefore, it is tempting to speculate that the known guard proteins may potentially be involved in the quality control of shuttling ncRNAs as well.

Moreover, Npl3, Gbp2, Hrb1, Hrp1, and Nab2 might not be the only guard proteins that surveil pre-mRNA maturation. Other factors might exist since quality control during processes such as RNA modifications has not yet been analyzed. For mRNAs, there is a wide array of post-translational modifications as well, including but not limited to methylation, acetylation, or pseudouridinylation. However, since 5′ capping, splicing, cleavage, and polyadenylation are the most common mRNA processing steps, these proteins represent the main facilitators of mRNA quality control.

It would be intriguing to explore whether similar mRNA quality control mechanisms exist in human cells. However, investigating these processes in humans presents significant technical challenges. In yeast, nuclear degradation can be blocked by knocking out a component of the nuclear exosome, and when combined with the knockout or mutation of a guard protein, this allows the study of faulty transcript leakage [16]. To our knowledge, a method that allows the specific inhibition of nuclear RNA degradation while enabling the investigation of RNA leakage has not yet been established in human systems. This lack of a suitable model for studying nuclear RNA surveillance limits our ability to directly explore these mechanisms in humans. As such, while insights from yeast studies provide a valuable foundation, further methodological advancements are necessary before analogous RNA quality-control systems in humans can be fully understood.

## Figures and Tables

**Table 1:** Overview of the currently established guard proteins ^1^.

	Npl3	Gbp2	Hrb1	Hrp1	Nab2
Alternative names	Nab1	Rlf7	Tom34	Nab4	-
	Mtr13			Nab5	
	Mts1			CFIB	
	Nop3				
Length	414 aa	427 aa	454 aa	534 aa	525 aa
Molecular weight	45.4 kDa	48.7 kDa	52.1 kDa	59.6 kDa	58.3 kDa
Median abundance (mol./cell)	34,936 ± 26,291	3171 ± 1870	5191 ± 1126	13,717 ± 8354	8716 ± 2946
Export receptor	Mex67	Mex67	Mex67	Mex67	Mex67
Import receptor	Mtr10	Mtr10	Mtr10	Kap104	Kap104
Essential in yeast?	No	No	No	Yes	Yes

^1^ aa: amino acids, mol./cell: molecules per cell.
